# Laser-activable murine ferritin nanocage for chemo-photothermal therapy of colorectal cancer

**DOI:** 10.1186/s12951-024-02566-6

**Published:** 2024-05-29

**Authors:** Jinmei Cheng, Jiaxin Li, Qilin Yu, Peishan Li, Junyi Huang, Jinhui Li, Leyang Guan, Zhiyong Xu, Jisheng Xiao, Xiaopin Duan

**Affiliations:** 1 Department of General Surgery, Zhujiang Hospital, Cancer Research Institute, School of Basic Medical Sciences, Southern Medical University, Guangzhou, 510515 Guangdong China; 2 Department of Cardiology, Heart Center, Guangdong Provincial Biomedical Engineering Technology Research Center for Cardiovascular Disease, Translational Medicine Research Center, Zhujiang Hospital, Southern Medical University, Guangzhou, 510280 China; 3 Experimental Education/Administration Center, School of Basic Medical Science, Southern Medical University, Guangzhou, 510515 China

**Keywords:** Murine ferritin nanocage, Thermal-responsive, Mitoxantrone, Chemo-photothermal therapy, Colorectal cancer

## Abstract

Chemotherapy, as a conventional strategy for tumor therapy, often leads to unsatisfied therapeutic effect due to the multi-drug resistance and the serious side effects. Herein, we genetically engineered a thermal-responsive murine Ferritin (mHFn) to specifically deliver mitoxantrone (MTO, a chemotherapeutic and photothermal agent) to tumor tissue for the chemotherapy and photothermal combined therapy of colorectal cancer, thanks to the high affinity of mHFn to transferrin receptor that highly expressed on tumor cells. The thermal-sensitive channels on mHFn allowed the effective encapsulation of MTO in vitro and the laser-controlled release of MTO in vivo. Upon irradiation with a 660 nm laser, the raised temperature triggered the opening of the thermal-sensitive channel in mHFn nanocage, resulting in the controlled and rapid release of MTO. Consequently, a significant amount of reactive oxygen species was generated, causing mitochondrial collapse and tumor cell death. The photothermal-sensitive controlled release, low systemic cytotoxicity, and excellent synergistic tumor eradication ability in vivo made mHFn@MTO a promising candidate for chemo-photothermal combination therapy against colorectal cancer.

**Graphical Abstract:**

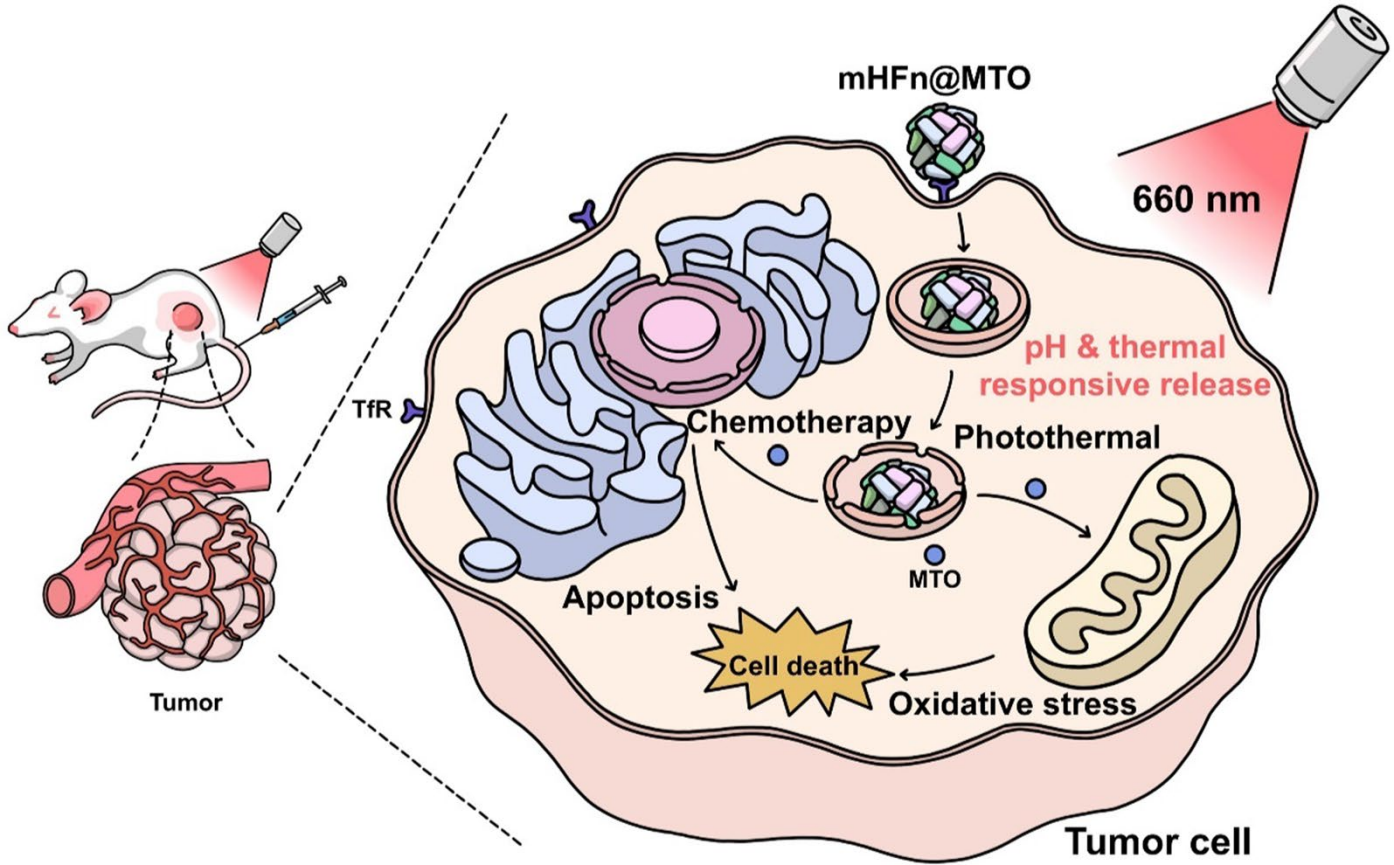

**Supplementary Information:**

The online version contains supplementary material available at 10.1186/s12951-024-02566-6.

## Introduction

Colorectal cancer (CRC) is a common gastrointestinal malignancy with high incidence and mortality rate worldwide [1]. Surgery combined with chemotherapy as the mainstay treatment for CRC patients, however, often leads to multi-drug resistance, as well as increased metastasis and recurrence [2–4]. Immunotherapy, although effective in a small subset (5% ~ 10%) of patients with deficient mismatch repair or microsatellite instability-high tumors [5, 6], has limited applicability to the overwhelming majority of CRC patients who are microsatellite stabilized.

Photothermal therapy (PTT) involves irradiation of light-absorbing agents accumulated in the tumor with light to convert optical energy into heat for thermal ablation of cancer cells. Theoretically, photothermal agents are non-toxic in the dark, but turn to be toxic to tumor cells upon the local applied light, leading to high treatment efficacy for all types of tumors with few side effects [7–12]. However, conventional photothermal therapy alone is not sufficient to completely eradicate tumors, leaving residual tumor margins and resulting in tumor recurrence. Therefore, combination strategies that integrate photothermal therapy with other therapies, such as chemotherapy [13, 14], immunotherapy [15, 16], and photodynamic therapy [17, 18], have emerged as promising approaches for treating various tumors.

Mitoxantrone (MTO), a broad-spectrum chemotherapeutic drug used in clinical settings [19, 20], also exhibits strong absorption in the wavelength range of 600–700 nm, making it an ideal candidate for chemo-photothermal combination therapy [21]. However, the nonspecific biodistribution, potential systemic toxicity, rapid clearance, and drug resistance severely limit its therapeutic efficiency [22, 23]. Therefore, novel vectors that enable specific targeting to and rapid release in tumor tissue would reduce systemic toxicity, slow drug resistance, and enhance therapeutic efficiency.

Protein nanocarriers, such as ferritin, hemoglobin, and albumin, have shown good biodegradability and biocompatibility, making them suitable for future clinical applications [24, 25]. Thanks to its unique nanocage structure and high binding affinity to CD71 (transferrin receptor 1, TfR1), human heavy chain ferritin (hHFn) has been extensively studied and applied in various areas [26, 27], such as drug delivery [28, 29], biocatalysis [30], optical therapy [31], medical imaging [32], and vaccine research [33, 34]. However, there is currently a lack of reports on murine ferritin. Herein, we firstly employed genetic engineering technique to construct a recombinant murine heavy chain ferritin (mHFn) and proved that mHFn possessed high affinity to TfR, which would facilitate the selective accumulation in tumor tissues but minimize the uptake by normal tissues following systemic administration. mHFn could assemble into nanocage with a dynamic size of ~ 10 nm in slightly alkaline solution and disassemble into monomers at acidic environment, endowing the pH-responsive drug release. mHFn also contains thermal-sensitive channels for the easy and effective encapsulation of MTO into its inner cavity (mHFn@MTO). Upon irradiation with a 660-nm laser, the photothermal effect of MTO caused the temperature to rise rapidly, then triggered the opening of channels, and finally led to the quick release of MTO in a thermal-responsive manner. The rapidly released MTO significantly induced the intracellular production and accumulation of reactive oxygen species (ROS) by integrating chemotherapy and photothermal therapy, thereby remarkedly causing tumor cells apoptosis in vitro and extraordinarily inhibiting tumor growth in vivo (Scheme 1). The pH- and thermal-responsive mHFn@MTO nanocage exhibited high tumor accumulation, good biosafety, and excellent anti-tumor effect, demonstrating its great potential for the chemo-photothermal combination therapy of colorectal cancer.

## Materials and methods

### Materials, cell lines, and animals

Mitoxantrone (> 97%), IPTG (Isopropyl β-D–Thiogalactopyranoside), and dialysis membranes (MWCO: 3500D) were bought from Solarbio (Beijing, China). Urea was obtained from J&K. (Beijing, China). Ni–NTA–Sefinose TM Column was sourced from Sangon (Shanghai, China). Ultracel-10 regenerated cellulose membrane was provided by Merck KGaA (Darmstadt, Germany). Other reagents were all purchased from Aladdin (Shanghai, China).

Murine embryonic fibroblast cells NIH3T3, murine colon adenocarcinoma cells CT26 and MC38 cells obtained from the American Type Culture Collection (ATCC, Rockville, MD) were cultured in RPMI 1640 and DMEM, respectively, supplemented with 10% fetal bovine serum (FBS, Gbico, Massachusetts, USA), 100 μg/mL streptomycin sulfate, and 100 Unit/mL penicillin G sodium. All cells were cultured in humidified incubator containing 5% CO_2_ at 37 °C.

Male C57BL/6 mice and male BALB/c mice (6 weeks, 18–22 g), provided by Guangdong Medical Laboratory Animal Center (Guangdong, China), were raised with sterile water and food in standard conditions. All ethical regulations relevant to animal research were approved by the Institutional Animal Care and Use Committee of Southern Medical University.

### Preparation and purification of mHFn

The recombinant plasmid pET-30a (+) of mHFn was firstly constructed, followed by the transform into *Escherichia coli* (Sangon, Shanghai, China). After incubation with 0.5 mM IPTG at 20 ℃ for 16 h, the cultured *E. coli* was collected, sonicated, and resuspended with 50 mM Tris–HCl containing 8 M urea and 300 mM NaCl (pH 8.0) at 4 ℃ for 1 h. The supernatant containing mHFn subunits after centrifugation at 12000 g for 10 min was then collected and purified by Ni–NTA–Sefinose (TM) Column according to the manufacturer’s instructions. Finally, the purified mHFn subunit was renatured and refolded to form mHFn nanocage by dialysis against the gradient urea from 8 to 0 M in Tris buffer. The purification and renaturation of mHFn were assessed by sodium dodecyl sulfate–polyacrylamide gel electrophoresis (SDS-PAGE) and UV-vis spectrophotometer (UV-2600, Shimadzu, Japan). The concentration of mHFn was detected by a bicinchoninic acid assay (BCA assay, GlpBio Technology, USA).

### Preparation and characterization of mHFn@MTO

To prepare mHFn@MTO, 0.18 mg of MTO was incubated with 4 mg mHFn in 50 mM Tris-HCl (pH 8.0) at 60 °C for 4 h, and then cooled to room temperature (25 °C). Free MTO was removed by ultra-centrifuging through an ultracel-10 regenerated cellulose membrane (MWCO: 10 kD). The hydrodynamic diameter, polydispersity index (PDI), and zeta potential of both mHFn and mHFn@MTO were determined by a dynamic light scattering instrument (DLS, Malvern, UK). The morphology of mHFn and mHFn@MTO nanocages was observed under a transmission electron microscope (TEM, Talos F200x, Thermo Fisher, USA). The characteristic absorption spectrum of mHFn@MTO was verified using a UV-Vis spectrophotometer.

To determine the loading of MTO, mHFn@MTO was first disassembled by HCl (20 mM, pH 2.0) and then denatured with methanol to extract the released MTO. The concentration of MTO was detected by high-performance liquid chromatography (HPLC, Agilent, USA) at the characteristic detection wavelength of 250 nm. The colloidal stability of mHFn@MTO nanocages in Tris or phosphate buffer solution (PBS) with or without 10% FBS at 37 °C was evaluated by monitoring the change in size and PDI for 24 h incubation.

### In vitro MTO release from mHFn@MTO

The particle sizes of mHFn@MTO under different pH and different of temperature were first recoded by DLS. Furthermore, the release profile of MTO from mHFn@MTO was evaluated in 0.1 M PBS (pH 7.4) and 0.1 M acetate buffer solution (ABS, pH 5.0) at 37 °C. Briefly, mHFn@MTO solution was sealed in a dialysis tube (MWCO: 3500 Da) and immersed in 5 mL release media with continuously shaking at 100 rpm. All release media were taken out at predetermined timepoints and replenished equal volume of fresh media. MTO concentration was measured using a UV-Vis spectrophotometer. Additionally, the release profile of MTO from mHFn@MTO at different temperatures was also assessed. Similarly, mHFn@MTO solution sealed in a dialysis bag was immersed in 5 mL Tris buffer and incubated at 40 °C, 50 °C, or 60 °C with continuously shaking at 100 rpm. At predetermined timepoints, release media were taken out for determination of MTO concentration.

### The affinity of mHFn@MTO to TfR

The expression of TfR in normal cells (NIH3T3) and tumor cells (CT26 and MC38) was first investigated by flow cytometry (FACS, LSRFortessa, BD). In brief, cells were incubated with primary anti-mouse TfR monoclonal antibody (dilution 1:100, Abcam, USA) for 1 h, followed by incubation with PE-conjugated secondary anti-mouse antibody (dilution 1:200, CST, USA) for another 30 min. Cells were then harvested, washed with PBS, and analyzed by FACS.

To validate the function of TfR in mediating the internalization of mHFn nanocage, cells seeded in confocal dishes  with a density of 2 × 10^5^ cells per dish were incubated with anti-mouse TfR antibody for 2 h, and then incubated with mHFn@MTO (MTO: 5 μg/mL) for an additional 4 h. Cells were then analyzed by FACS or stained with Hochest 33342 for observation under confocal laser scanning microscope (CLSM, Olympus FV-1000, Japan).

### Photothermal effect of mHFn@MTO

mHFn@MTO solution at various concentrations (40, 60, 80, and 100 μg/mL) was irradiated with a 660 nm laser at different power densities (0.3, 0.4, 0.5 W/cm^2^), the temperature changes were recorded using a FLIR infrared thermal camera (Teledyne FLIR, USA). The photothermal effect of different groups (MTO: 100 µg/mL) was also analyzed at a power density of 0.5 W/cm^2^. The photothermal stability of MTO and mHFn@MTO was evaluated after six cycles of irradiation for 10 min and cooling for 10 min, the temperature changes during irradiation-cooling cycles were recorded and plotted to evaluate the stability.

### Laser-responsive release of mHFn@MTO

mHFn@MTO (100 μg/mL) was irradiated with different power densities of 660 nm laser (0.3, 0.4, and 0.5 W/cm^2^) for 5 min, and then ultra-centrifugated to separate the released MTO. The concentration of MTO in the collected samples was measured using a UV-Vis spectrophotometer. Furthermore, the release behavior of mHFn@MTO during cycles of irradiation for 5 min and cooling for 5 min was also investigated.

### Cellular uptake and subcellular localization

CT26 cells were seeded in 6-well plates at a density of 2 × 10^5^ cells per well and allowed to attach overnight. Cells were then incubated with free MTO or mHFn@MTO at a concentration of 10 μg/mL for 1, 2, or 4 h. At predetermined timepoints, cells were collected, washed three times with PBS, and analyzed using a flow cytometry system. For subcellular localization, CT26 cells were seeded on 10 mm^2^ glass coverslips that were placed in six-well plates and treated as mentioned above. After staining lysosomes with LysoTracker Green and nucleus with Hoechst (all from Beyotime, China), cells were observed and photographed using CLSM..

### In vitro cytotoxicity

CT26 cells and MC38 cells were seeded in 96-well plates at a density of 3 × 10^3^ cells/well and incubated with of MTO or mHFn@MTO at various concentrations for 4 h. Then, cells were irradiated with a 660 nm laser at a power density of 0.5 W/cm^2^ for 5 min and grew for another 48 h. Cell viability was measured using a CCK-8 assay according to the product specifications.

### ROS accumulation

CT26 cells seeded in 6-well plates (2 × 10^5^ cells/well) were incubated with MTO or mHFn@MTO at a MTO concentration of 1 μg/mL for 4 h and irradiated with 660 nm laser (0.5 W/cm^2^) for 5 min. Cells were then collected, stained with 10 μM DCFH-DA (Beyotime, China) for 30 min, washed with PBS for three times, and detected by flow cytometry. The intracellular ROS accumulation in CT26 cells was further observed by an inverted fluorescence microscope (Nikon ECLIPSE Ti2, Japan).

### Mitochondrial membrane potential assay

CT26 cells were treated as mentioned above and stained with JC-1 fluorescent probe (Beyotime, China) at a concentration of 1 μg/mL for 20 min in the dark. Subsequently, cells were collected, washed with PBS, and analyzed by flow cytometry. The shift of JC-1 fluorescence from red (aggregates, normal mitochondrial membrane potential) to green (monomer, lowered mitochondrial membrane potential) was further observed using an inverted fluorescence microscope.

### Live/dead cell staining

CT26 cells were incubated with MTO or mHFn@MTO (MTO: 1 μg/mL) for 4 h, irradiated with a laser at a power density of 0.5 W/cm^2^ for 5 min, and then cultured for an additional 24 h. Treated cells were stained with Calcein-AM/pyridine iodide (PI) cell viability assay kit (Beyotime, China) for 30 min in the dark at 37 ℃, washed three times with PBS, and observed under inverted fluorescence microscope.

### Cell apoptosis and cell cycle assay

CT26 cells were incubated with MTO or mHFn@MTO (MTO: 1 μg/mL) for 4 h and irradiated with a laser at a power density of 0.5 W/cm^2^ for 5 min. Treated cells were harvested after 24 h, washed with ice-cold PBS, and stained with Annexin V-FITC and PI (Beyotime, China) for 30 min in the dark at 37 ℃, followed by the analysis of flow cytometry. For cell cycle assay, treated cells were collected, fixed with 70% cold ethanol, treated with DNA staining solution (containing RNase A) and permeabilization solution (containing PI) for 30 min according to the product specification. The alteration of cell cycle was finally detected by flow cytometry.

### In vivo tumor targeting and penetration

CT26 tumor-bearing mice models were established by subcutaneously injecting 1 × 10^6^ tumor cells into the right flank of BALB/c mice. mHFn@MTO at MTO dose of 4 mg/kg was intravenously injected into mice as the tumor volume reached ~ 100 mm^3^. The biodistribution of mHFn@MTO in tumor-bearing mice were observed by an IVIS Imaging System (IVIS Lumina II, Caliper Life Science, USA) at predetermined timepoints (1, 2, 4, 8, 12, 24 and 48 h). The major organs (heart, liver, spleen, lung, kidney, and tumor) were excised at 8 h, 24 h and 48 h post administration, photographed under the IVIS Imaging System, and then homogenized for MTO quantification by multi-mode microplate reader (BioTek Synergy HTX, USA) with excitation at 610/20 nm and emission at 680/20 nm.

To evaluate the effect of laser irradiation on tumor penetration of mHFn@MTO, CT26 tumor-bearing mice were intravenously injected with mHFn@MTO (MTO: 4 mg/kg) and irradiated 8 h post injection with laser for 5 min at a power density of 0.5 W/cm^2^. At 24 h post irradiation, the main organs were excised and photographed under the IVIS Imaging System to observe the effect of laser irradiation on tumor accumulation. Tumors were further sectioned, stained with anti-CD31 or anti-HIF-1α antibodies (1:400, Abcam, USA), and observed under CLSM.

### In vivo photothermal effect and anticancer therapeutic efficacy

CT26- or MC38-tumor bearing mice were randomly divided into six groups (n = 6), and intravenously injected with PBS, MTO, or mHFn@MTO at an equivalent MTO dosage of 4 mg/kg. 8 h after administration, three laser groups were irradiated with a 660 nm laser for 5 min at a power density of 0.5 W/cm^2^. The temperature changes in irradiated tumor sites were recorded using a FLIR infrared thermal camera to assess the photothermal effect. Tumors were measured every 2 days and calculated using the following formula: volume = (length × width^2^)/2. Body weights of the mice were also monitored every 2 days to evaluate the toxicity of formulations. At the endpoint of experiments, mice were sacrificed, tumors were weighted, sectioned, stained with H&E, anti-Ki67 antibody (Abcam) or TUNEL, and observed under CLSM. Major organs were also collected, fixed, embedded in paraformaldehyde, sectioned, and stained with H&E. Blood was collected from MC38 tumor-bearing mice after sacrificing them. Blood biochemical analysis was further conducted to determine the levels of alanine aminotransferase (ALT), aspartate aminotransferase (AST), alkaline phosphatase (AKP), creatinine (CRE), and urea nitrogen (BUN).

### Statistical analysis

All data were presented as mean ± SD from at least 3 independent experiments of biological replicates, the specific sample size (n) was illustrated in the figure legend. GraphPad prism 9 was used to analyze the statistical significance by two-tailed unpaired t-test. The difference was regarded as significant when the p value was less than 0.05 (* *P* < 0.05, ** *P* < 0.01, *** *P* < 0.001, and **** *P* < 0.0001).

## Results and discussion

### Preparation and characterization of mHFn@MTO

**Fig. 1:**
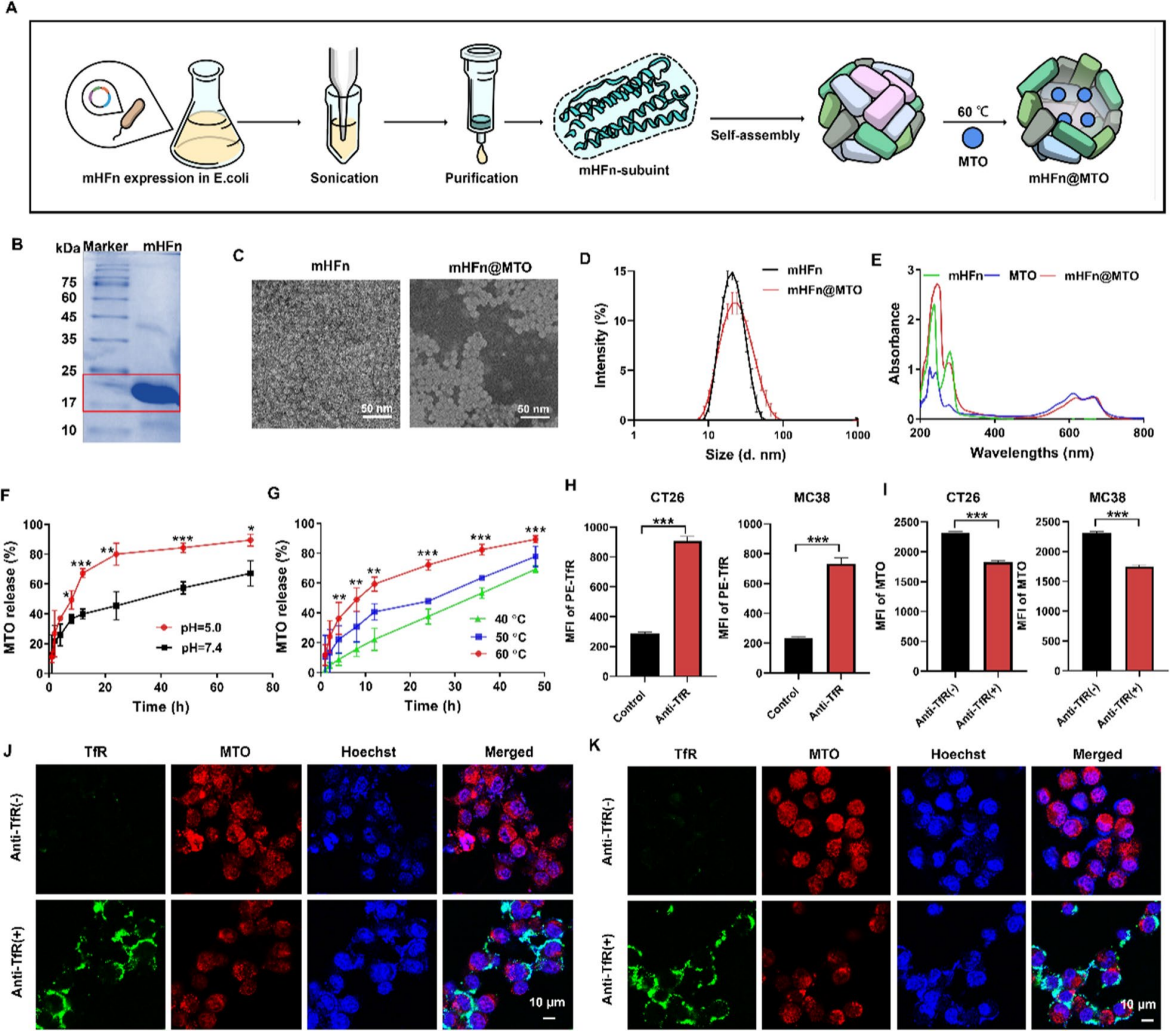
Characterization of the pH- and thermal-responsive mHFn@MTO nanocage. **A** The preparation process of mHFn@MTO nanocage. **B** SDS-PAGE characterization of mHFn protein after purification. **C** TEM images of mHFn and mHFn@MTO nanocages. **D** Hydrodynamic sizes of mHFn and mHFn@MTO nanocages measured by DLS. **E** The absorption spectra of mHFn, MTO, and mHFn@MTO nanocage, determined using a UV-vis spectrophotometer. **F**, **G** Cumulative release of MTO from mHFn@MTO nanocage at (**F**) different pH and (**G**) different temperature. **H** The expression of TfR in CT26 and MC38 cells. **I** The cellular uptake of mHFn@MTO in (**I** and **J**) CT26 and (**I** and **K**) MC38 cells in the presence or absence of anti-TfR, determined by (**I**) flow cytometry and (**J** and **K**) CLSM. The data are represented as mean ± SD (n = 3). *P < 0.05, **P < 0.01, ***P < 0.001

mHFn was successfully constructed and expressed in *Escherichia coli*, followed by purification using a Ni–NTA-Sefinose Column. MTO was easily encapsulated into the inner cavity of mHFn through thermal treatment (60 ℃ for 4 h) to obtain mHFn@MTO, with encapsulation efficiency of 90.32 ± 4.67% and drug loading of 9.33 ± 0.01 wt% respectively (Fig. 1A), consistent with previous research on human ferritin-based nanocages [27]. SDS-PAGE confirmed the molecular weight of mHFn to be approximately 22 kDa (Fig. 1B). TEM revealed the well-defined spherical structure of mHFn with an inner cavity, which disappeared upon MTO encapsulation, however, mHFn@MTO nanocage still maintained the good mono-dispersity after drug loading (Fig. 1C). The DLS determination revealed that mHFn showed no obvious change on the hydrodynamic size (22.09 ± 0.69 nm *vs* 21.17 ± 0.46 nm) and the PDI (0.27 ± 0.06 *vs* 0.23 ± 0.03) before and after MTO loading (Fig. 1D, and Fig. S1A), whereas the zeta potential slightly increased from ~ -18 mV to ~ -10 mV (Fig. S1B), indicating that the drug loading did not significantly affect the properties of mHFn.

The UV-vis absorption spectrum of mHFn@MTO showed the characteristic absorption peaks at 280 nm for protein and 665 nm for MTO, confirming the successful loading of MTO into the inner cavity of mHFn (Fig. 1E). Additionally, mHFn@MTO exhibited remarkable stability in both Tris and PBS containing 10% FBS, as evidenced by no significant changes in size, PDI and zeta potential over a period of 24 h (Fig. S2).

### mHFn@MTO releases MTO in pH- and thermal-responsive manner

Since the tumor microenvironment is relatively acidic, we first observed the particle size changes of mHFn@MTO under different pH condition. The results showed that the particle size of mHFn@MTO gradually increased as the pH decreased from 8.0 to 5.0, and exhibited obvious protein turbidity when the pH decreased to 2.0 (Fig. S3A). Furthermore, the release of MTO from nanocage in response to different pH was investigated by dialysis method. As the pH decreased from 7.4 to 5.0, the release of MTO increased from 45.35 ± 9.46% to 80.06 ± 7.41% after 24 h incubation, which is almost 2 times increase (Fig. 1F). The pH-dependent release of MTO is advantageous for anti-tumor therapy as it minimizes drug release in the bloodstream and ensures an adequate amount of drug reaches the tumor tissue, facilitating efficient killing of tumor cells upon internalization of mHFn@MTO. The pH sensitivity of mHFn could be attributed to the pH-mediated disassembly/reassembly, in which the salt bridges and hydrogen bonds between protein dimers under extreme acidic or alkaline conditions are lost, leading to reversible disassembly and reassembly of protein subunits [35–37]. Additionally, the release of MTO from mHFn nanocage was also thermal-responsive, as the release after 12 h incubation significantly increased from 22.17 ± 7.47% to 40.60 ± 5.46% and 59.28 ± 4.72% when the temperature elevated from 40 °C to 50 °C and 60 °C, respectively (Fig. 1G). mHFn@MTO still remained intact at temperature as high as 60 °C (Fig. S3B), suggesting that the high MTO release at 60 °C is due to the “opening” of the channels on mHFn that would facilitate the release of MTO.

### mHFn@MTO shows high affinity to TfR

As an endogenous ligand of TfR, mHFn theoretically can specifically target tumor cells through binding to the highly expressed TfR on tumor cells surface [38, 39]. We first investigated the expression of TfR in normal cells (NIH3T3 cells) and colorectal cancer cells (CT26 and MC38 cells) by flow cytometry. The results showed that the expression of TfR in tumor cells was significantly upregulated compared to normal cells (Fig. 1H, and Fig. S4). To further validate the affinity of mHFn to TfR, we used specific monoclonal antibody to block the TfR and observed the internalization of mHFn@MTO by different cells. The fluorescence intensities of MTO in both CT26 and MC38 cells were significantly reduced after the blockade of TfR (Fig. 1I), while the uptake of mHFn@MTO by NIH3T3 cells was not obviously affected by TfR blockade (Fig. S5A). Moreover, the CLSM images showed the same trend as flow cytometry, revealing higher TfR expression on tumor cells and reduced internalization of mHFn@MTO in tumor cells when TfR was blocked (Fig. 1J, K and Fig. S5B).

### Photothermal effect enhances the rapid release of MTO from mHFn@MTO

**Fig. 2:**
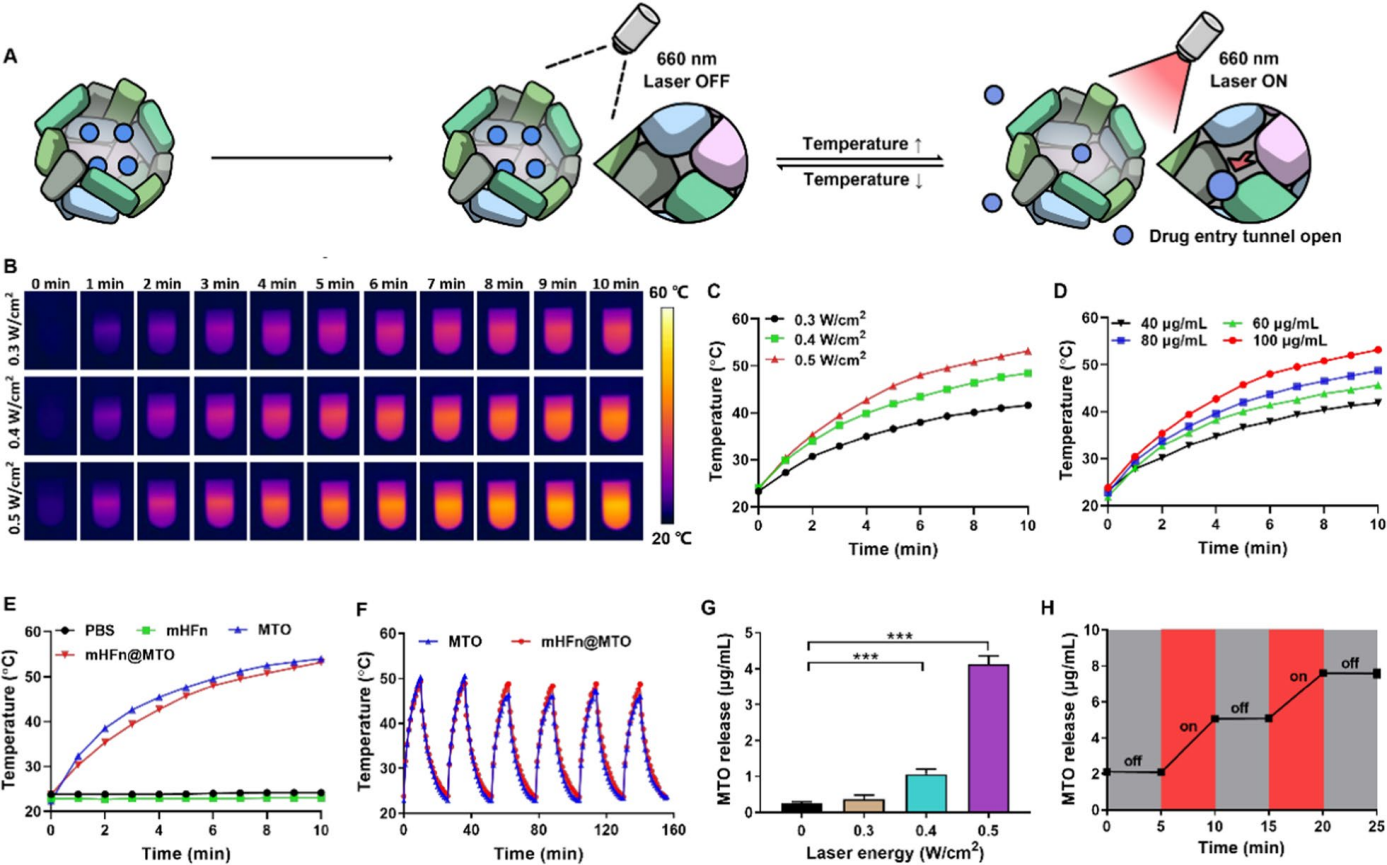
Photothermal effect enhances MTO release. **A** The scheme showing the laser-responsive release process of MTO from mHFn@MTO nanocage. **B**, **C** Photothermal performance of mHFn@MTO after 660-nm laser irradiation with different power densities. **D** Photothermal effect of mHFn@MTO at different MTO concentrations after laser irradiation. **E** Photothermal heating curves of PBS, mHFn, MTO, mHFn@MTO under 660 nm laser irradiation (MTO: 100 μg/mL). **F** Photothermal stability of MTO and mHFn@MTO exposed to 660-nm laser. **G** The in vitro MTO release from the mHFn@MTO with different laser intensities. **H** The in vitro release profile of MTO from mHFn@MTO with a cyclic variation of the laser between on and off for several repetitions. The data are represented as mean ± SD (n = 3). ***P < 0.001

We further investigated whether the photothermal effect of MTO could enhance its release. Theoretically, MTO should be able to be released through the opened thermal-sensitive channels upon laser irradiation due to the raised temperature by photothermal effect, while the release of MTO should slow to a halt when the laser was turned off (Fig. 2A). We first confirmed the photothermal properties of mHFn@MTO in an aqueous solution, as the temperature gradually increased with the increment of laser power intensity and MTO concentration upon 660 nm laser irradiation (Fig. 2B–D). Specifically, the temperature of free MTO and mHFn@MTO solution both reached approximately 50 °C at a concentration of 100 μg/mL MTO under irradiation with 0.5 W/cm^2^, indicating the comparable photothermal effect of mHFn@MTO to free MTO (Fig. 2E). Furthermore, the highest temperature that can be reached by mHFn@MTO kept consistent during six repeated heating/cooling cycles by turning the laser on/off, while the highest temperature of MTO slightly decreased with each cycle (Fig. 2F), demonstrating that mHFn@MTO exhibits higher photothermal stability compared to free MTO. These results verified that the encapsulation of MTO into mHFn inner cavity not only had no influence on the photothermal performance, but also promoted the photothermal stability.

We found a burst release of MTO from mHFn@MTO with the laser turned on, while almost no MTO was detected in the control group without laser irradiation. In addition, the release of MTO significantly accelerated with increased laser power intensities from 0.3 to 0.5 W/cm^2^ (Fig. 2G), suggested that the release of MTO could be significantly promoted with the assistance of an externally triggered temperature increase response to laser irradiation. The release characteristic of MTO measured as the laser was cyclically turned on and off every 5 min also showed laser-responsiveness, as the leakage of MTO increased within 5 min when the laser was turned on, whereas almost no MTO was released when the laser was off (Fig. 2H). Overall, mHFn@MTO was determined to be an excellent photothermal-responsive nanocage capable of achieving rapid release of MTO under laser irradiation.

### mHFn@MTO effectively kill tumors by chemo-photothermal effect

**Fig. 3:**
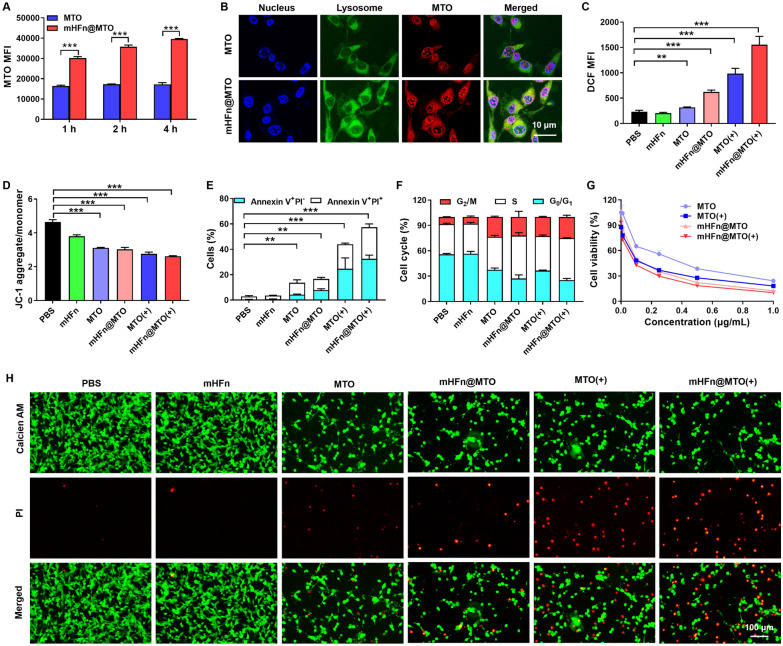
mHFn@MTO effectively kills tumor cells. **A** Kinetic uptake of MTO and mHFn@MTO in CT26 cells at 1, 2, 4 h, respectively. **B** Subcellular localization of free MTO and mHFn@MTO in CT26 cells after 4 h incubation, observed by CLSM. **C**, **D** Quantification of (**C**) total ROS accumulation and (**D**) mitochondrial membrane potential in CT26 cells after different treatments. **E, F** Quantification of (**E**) cell apoptotic percentage and (**F**) cell cycle of CT26 cells incubated with different formulations. **G** Viability of CT26 cells after incubation with various concentrations of MTO and mHFn@MTO with or without laser irradiation. **H** Live/dead staining images of CT26 cells with different treatments. Live cells were stained green with calcein-AM, while dead cells were stained red with PI. The data are represented as means ± SD (n = 3). **P < 0.01 and ***P < 0.001 by a two-tailed unpaired t-test

We first investigated the internalization of mHFn@MTO on CT26 cells after incubation for 1, 2, or 4 h. The intracellular fluorescence intensity of free MTO reached its maximum within 1 h and did not increase further, indicating the rapid diffusion of free MTO to tumor cells. In contrast, the fluorescence intensities of mHFn@MTO in cells gradually increased over time, and were all much higher than that of free MTO at all tested timepoints (Fig. 3A), indicating the high internalizing ability of mHFn@MTO. CLSM also confirmed the preferential intracellular uptake of mHFn@MTO compared to free MTO (Fig. 3B and Fig. S6, S7). Additionally, colocalization of mHFn@MTO with lysosomes was observed, suggesting that mHFn@MTO might undergo energy-consuming endocytosis.

The higher level of ROS in tumor cells due to highly active metabolism makes themselves more sensitive to intracellular ROS accumulation compared to normal cells [40, 41]. Treatment with free MTO alone slightly increased the intracellular ROS level compared to PBS or single mHFn group. However, when combined with laser irradiation, a notable increase in ROS generation was observed, reaching 4.2-fold higher than the PBS group. Remarkably, the mHFn@MTO with irradiation showed the highest ROS accumulation, achieving 6.7-fold higher than the PBS group (Fig. 3C and Fig. S8). This substantial increase in ROS levels can cause cell death by damaging the cells membrane, proteins, DNA, and other cellular components [42, 43]. The effect of ROS on mitochondrial membrane potential was first assessed using JC-1 probe. Intact mitochondria with high potential exhibits red fluorescence due to the aggregation of JC-1 within the mitochondria, while damaged mitochondrial with low potential shows green as JC-1 released to cytoplasm and dissociated to monomers. As expected, a significant reduction in red fluorescence and an evident increase in green fluorescence were observed after treatment with mHFn@MTO plus irradiation (Fig. 3D and Fig. S9, S10), indicating its ability to cause mitochondrial damage.

The decrease in mitochondrial membrane potential is a characteristic event in early apoptosis [44]. We found that free MTO and mHFn@MTO without laser irradiation induced apoptosis on a small number of cells, resulting in 13.61 ± 2.26% and 16.61 ± 2.28% apoptotic cells, respectively. By contrast, a significant increase in apoptotic cells was observed on free MTO and mHFn@MTO with laser irradiation, leading to 43.97 ± 7.58% and 57.43 ± 1.30% of cells undergoing apoptosis, respectively, suggesting the highest ability of mHFn@MTO plus laser to induce cell apoptosis (Fig. 3E and Fig. S11). Similarly, mHFn@MTO plus laser showed the highest capability to alter the cell cycle distribution, causing a decrease in the percentage of cells in the G_0_/G_1_ phase and an increase in the percentage of cells in the S and G_2_/M phase (Fig. 3F and Fig. S12). Our results were consistent with previous findings that cells in the late S phase are the most sensitive to MTO-induced cell death, although MTO is reported to be capable of killing cancer cells at any stage of the cell cycle [45, 46].

Due to the multiple effects on cells, MTO exhibited relatively high cytotoxicity against murine colorectal adenocarcinoma CT26 cells with IC_50_ values of 0.32 ± 0.04 μg/mL. When combined with laser irradiation, MTO showed even higher cytotoxicity, evidenced by the decreased IC_50_ to 0.13 ± 0.02 μg/mL, suggesting the synergistic effect of chemo-photothermal therapy. Interestingly, mHFn@MTO demonstrated much stronger inhibition on CT26 tumor cells than free MTO, with IC_50_ values of 0.12 ± 0.01 μg/mL, which further decreased to 0.08 ± 0.01 μg/mL when combined with laser irradiation (Fig. 3G and Table S1). The same trend was also observed on MC38 cells, confirming the powerful tumor killing effect of mHFn@MTO with irradiation (Fig. S13 and Table S1). In contrast, no obvious cytotoxic effects were observed for mHFn, even at a relatively high concentration of 250 μg/mL (Fig. S14), suggesting the biosafety of mHFn protein as a novel drug delivery vehicle. The remarkable tumor cell killing effect of mHFn@MTO plus laser was further confirmed by Calcein-AM/PI staining. The results revealed that the PBS and mHFn groups had minimal cell death, while all other groups containing MTO exhibited higher efficacy in killing CT26 cells, especially when combined with laser irradiation (Fig. 3H). Notably, more than half of the CT26 tumor cells were killed after treatment with mHFn@MTO plus laser, demonstrating the potent tumor cell elimination ability achieved through the synergy of photothermal-chemotherapy.

### mHFn@MTO facilitates tumor accumulation and penetration

**Fig. 4:**
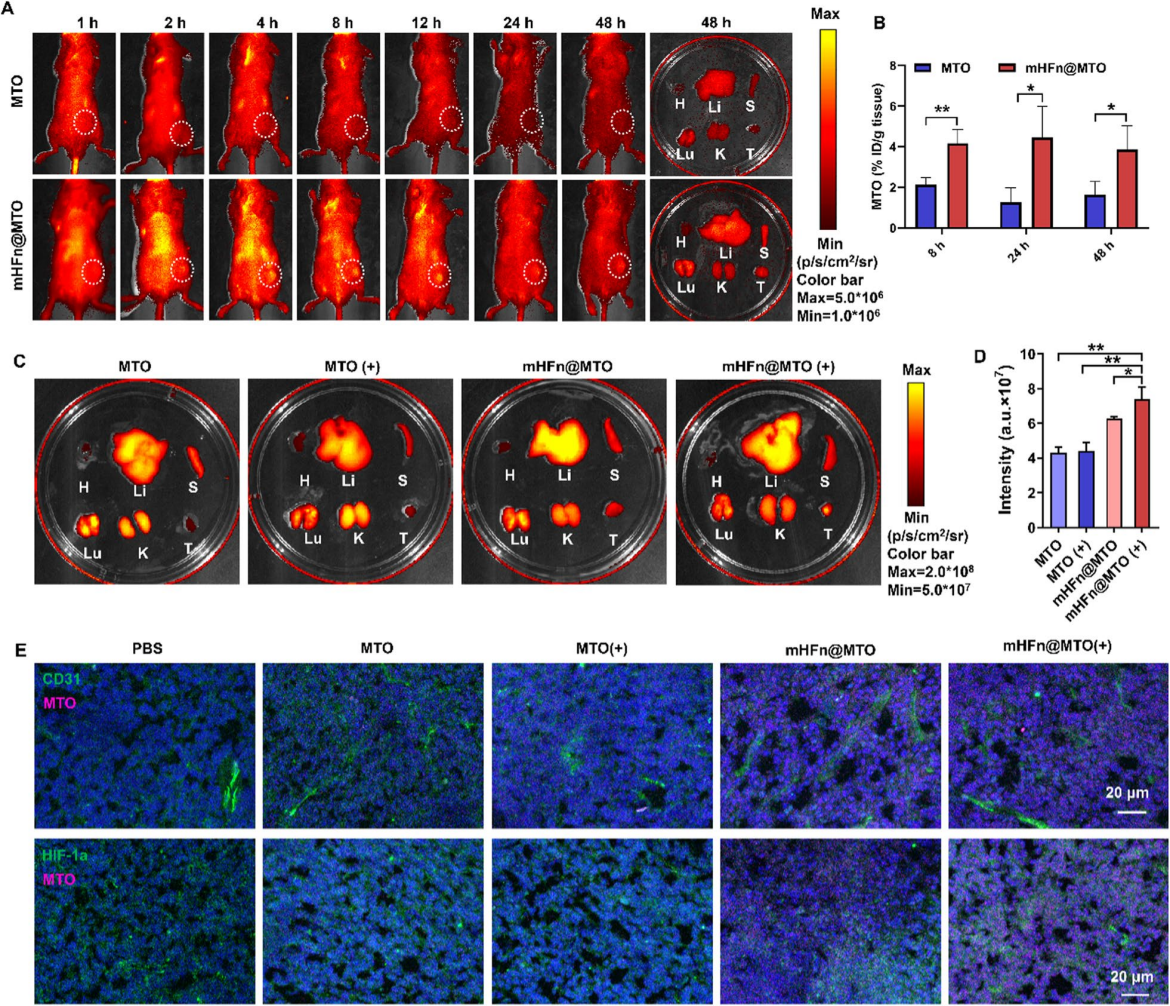
mHFn@MTO facilitates tumor accumulation and penetration. **A** In vivo fluorescence imaging of CT26 tumor-bearing BALB/c mice at predetermined timepoints following intravenous injection of free MTO and mHFn@MTO at MTO dose of 4 mg/kg. **B** Quantification of mHFn@MTO accumulation in excised tumors at 8 h, 24 h and 48 h. **C** Fluorescence imaging of excised organs and tumors in CT26 tumor-bearing BALB/c mice at 24 h post laser irradiation. **D** Quantitative fluorescence intensity of MTO and mHFn@MTO in tumors at 24 h post irradiation. **E** Immunofluorescent staining of CT26 tumors showing the colocalization of MTO with tumor vessels (stained with anti-CD31) and hypoxic tumor tissues (stained with anti-HIF-1α). The data are represented as means ± SD (n = 3). *P < 0.05 and **P < 0.01 by a two-tailed unpaired t-test

The tumor targeting ability of mHFn was investigated in CT26 tumor-bearing mice by intravenously injecting mHFn@MTO and monitoring the fluorescence intensities of MTO at predetermined timepoints using an IVIS Imaging System. Free MTO was rapidly cleared from the body and showed relatively low accumulation in the tumor tissue. In contrast, mHFn@MTO gradually accumulated in the tumor at 2 h post-administration and remained there for a longer period, as shown by high level of intratumoral fluorescence intensity even at 48 h post-administration (Fig. 4A). This enhanced and prolonged accumulation can be attributed to the intrinsic binding ability of mHFn to TfR that allows for efficient tumor targeting. The ex vivo quantification of MTO content in collected tumor tissues further confirmed the higher accumulation of mHFn@MTO than free MTO in tumor sites at all tested timepoints, and the accumulation of mHFn@MTO showed maximum at 8 h post-administration, lasted to 24 h, and then began to reduce slightly at 48 h (Fig. 4B and Fig. S15).

The effect of laser irradiation on in vivo penetration of mHFn@MTO into deep tumor regions was further investigated in CT26 tumor-bearing mice. The fluorescence signals from free MTO with or without laser irradiation were both difficult to observe in the tumors 24 h after administration, likely due to its short blood half-life and poor targeting ability. On the contrary, mHFn@MTO exhibited much higher tumor accumulation compared to free MTO, and the tumor accumulation was further enhanced by laser irradiation (Fig. 4C, D). The immunofluorescent staining also showed that combining mHFn@MTO with laser irradiation enabled the nanocages to diffuse further away from the blood vessels and penetrate deeper into the tumor (Fig. 4E). The high ability of mHFn@MTO with irradiation to penetrate the deep tumor regions might partly be due to the small particle size that facilitates penetration. In addition, the photothermal effect would also enhance tumor tissue penetration by increasing tumor blood flow [47, 48]. The mHFn@MTO combined with laser also exhibited the highest accumulation in hypoxic tumor tissues, as indicated by the colocalization of MTO fluorescence with HIF-1α (Fig. 4E). These findings suggest that mHFn@MTO plus laser facilitates the penetration of MTO into deeper hypoxic tumor regions, which is beneficial to enhance its effectiveness in killing tumor cells.

### mHFn@MTO promotes chemo-photothermal effects in vivo

**Fig. 5:**
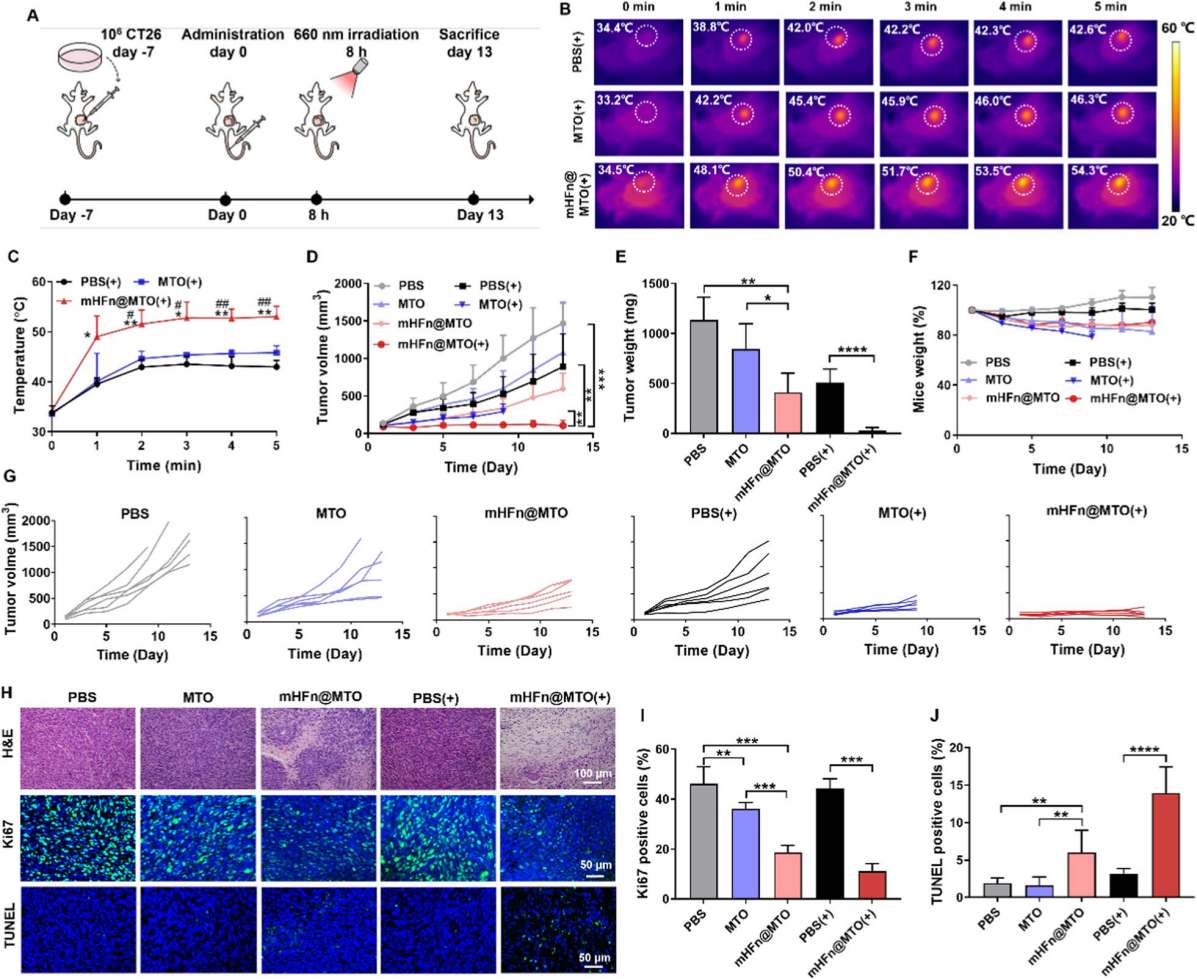
mHFn@MTO promotes chemo-photothermal effects on CT26 tumors. **A** Experimental design for the treatment of CT26 tumor-bearing mice. **B** Photothermal images and **C** quantification of temperature changes in different groups after laser irradiation for 5 min [*: mHFn@MTO(+) *vs* PBS(+), #: mHFn@MTO(+) *vs* MTO(+)]. **D** Growth curves of CT26 tumors after different treatments. **E** Tumor weight of different treatment groups at the end of experiment. **F** Body weight changes during treatment. **G** Individual tumor growth curves in response to different treatments. **H** H&E staining, Ki67 immunofluorescent staining, and TUNEL assay of tumors after different treatments. **I, J** Quantification of (**I**) Ki67 and (**J**) TUNEL positive cells in tumor tissue. The data are represented as means ± SD (n = 6). *P < 0.05, **P < 0.01, ***P < 0.001, and ****P < 0.0001 by a two-tailed unpaired t-test

The chemo-photothermal effect was investigated on CT26 tumor-bearing mouse models that were established by injecting 1 × 10^6^ cells subcutaneously into the right flanks of BALB/c mice. Once the tumor volume reached approximately 100 mm^3^, the tumor-bearing mice were randomly divided into six groups, intravenously injected with PBS, MTO or mHFn@MTO at a dose of 4 mg MTO/kg, and irradiated for 5 min at dose of 0.5 W/cm^2^ on 8 h after drugs administration (Fig. 5A). mHFn@MTO with irradiation significantly increased the temperature to 53.0 ± 2.1 °C, which was much higher than that of MTO (45.8 ± 1.4 °C) and PBS (43.0 ± 1.3 °C) with irradiation (Fig. 5B, C). MTO itself showed very limited inhibitory effect on tumor growth (602.8 ± 233.9 mm^3^ on day 9), compared to PBS group (1053.4 ± 316.0 mm^3^ on day 9), while mHFn@MTO significantly inhibited the tumor volume to 336.8 ± 133.4 mm^3^ (Fig. 5D, E), which possibly could be attributed to the tumor-targeting ability of mHFn nanocage that enhances the delivery of MTO to tumor site. When combined with laser irradiation, the inhibitory of MTO on tumor growth obviously increased with tumor volume of 291.3 ± 102.9 mm^3^ on day 9. However, MTO plus laser resulted in an aggressive decrease in the mice's body weight, and the mice had to be euthanized on day 9 due to ethical reasons in accordance with animal protocols at Southern Medical University (Fig. 5F). The severe weight loss in MTO plus laser group might have resulted from the systemic toxicity caused by the non-specific distribution of free MTO and the potential inflammatory response induced by PTT [49, 50]. Due to the effective photothermal effect, mHFn@MTO combined with laser showed the strongest inhibition on tumor growth, retarding tumor volume to 114.7 ± 39.5 mm^3^ on day 9 and completely eradicating 2/6 CT26 tumors on day 13 (Fig. 5G), highlighting the potent therapeutic efficacy of mHFn@MTO when combined with laser irradiation. Consistent with the tumor growth curve, the tumors treated with mHFn@MTO plus laser had the smallest weights on day 13 (Fig. 5E), further demonstrating the excellent anti-tumor effect achieved in vivo by mHFn@MTO-mediated chemo-photothermal therapy. Interestingly, mHFn@MTO plus laser led to negligible body weight loss even if it may also cause certain inflammatory response, thanks to its tumor-targeting capability. The excised tumors were further sectioned for histopathological examination and immunofluorescent staining. H&E staining and TUNEL assay revealed that mHFn@MTO with irradiation caused the most damage and induced the most apoptosis to tumor tissue, compared to other treatments (Fig. 5H, I). Immunofluorescence staining with Ki67 demonstrated that mHFn@MTO combined with irradiation also effectively inhibited tumor cell proliferation (Fig. 5H, J). These results further confirmed the extraordinary anti-tumor effect of mHFn@MTO-mediated chemo-photothermal therapy.

**Fig. 6:**
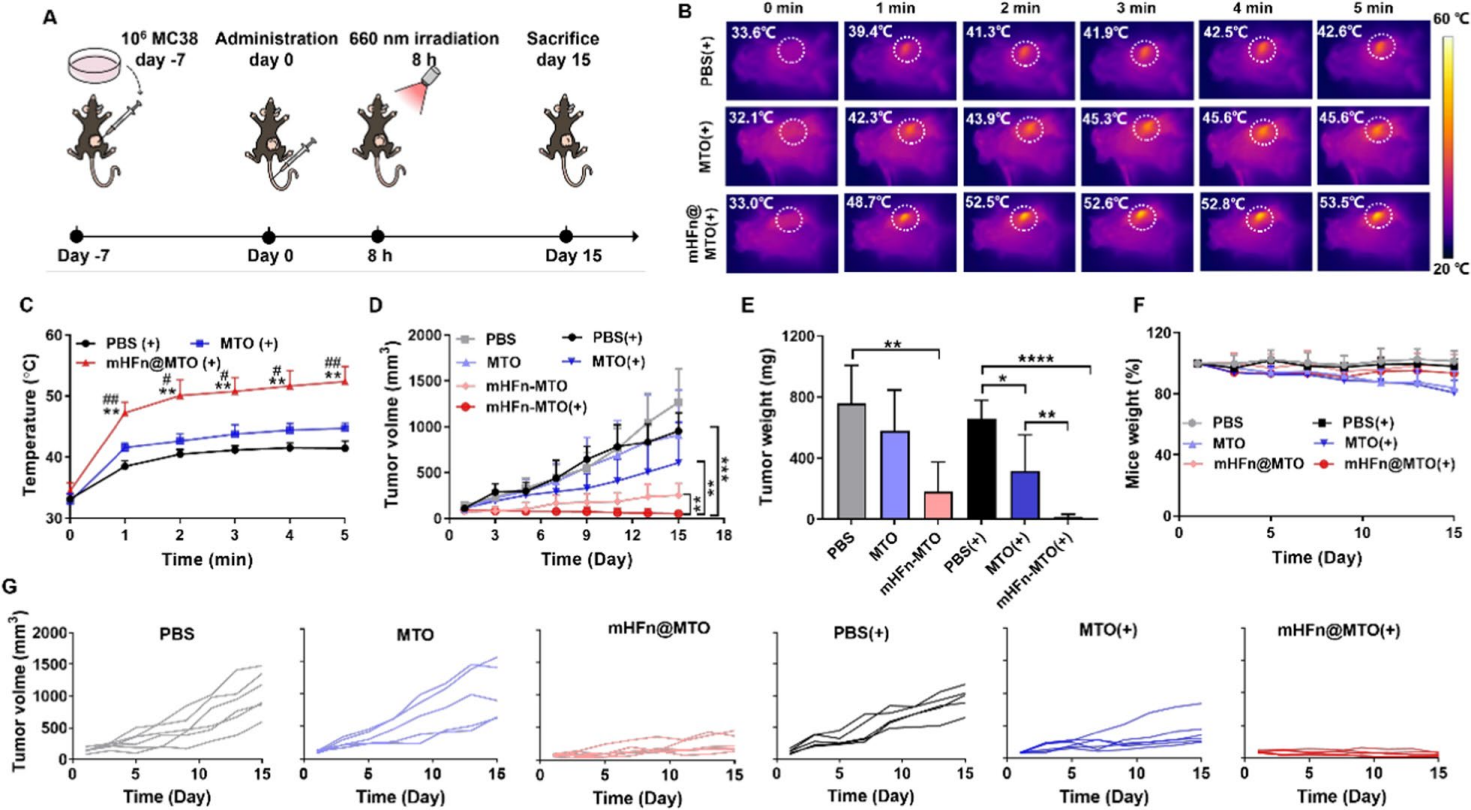
mHFn@MTO enhances chemo-photothermal effects on MC38 tumors. **A** Experimental design for the treatment of MC38 tumor-bearing mice. **B** Photothermal images and **C** quantification of temperature changes with laser irradiation for 5 min. [*: mHFn@MTO(+) *vs* PBS(+), #: mHFn@MTO(+) *vs* MTO(+)] **D** Growth curves of MC38 tumors after different treatments. **E** Tumor weights at the end of treatment. **F** Body weight changes during treatment. **G** Individual tumor growth curves of MC38 tumors in response to different treatments. The data are represented as means ± SD (n = 6). *P < 0.05, **P < 0.01, ***P < 0.001, and ****P < 0.0001 by a two-tailed unpaired t-test

The enhanced chemo-photothermal effect was further confirmed on MC38 tumor-bearing mouse models (Fig. 6A). Similarly, mHFn@MTO had a higher photothermal effect than free MTO due to the higher tumor accumulation, increasing the temperature at tumor site to 52.0 ± 2.3 °C, compared to 44.7 ± 0.9 °C of MTO group (Fig. 6B, C). As a result, mHFn@MTO plus laser treatment displayed an extraordinary antitumor effect and even led to the complete eradication of 2/6 MC38 tumors at day 15, by contrast, MTO with irradiation only showed moderate inhibition on tumor growth (Fig. 6D, G). Although MTO alone or combined with laser also caused weight loss of MC38 tumor-bearing mice due to the non-specific distribution and the potential inflammatory response, the weight loss caused by MTO plus laser on MC38 tumor-bearing mouse was much less than that on CT26 tumor-bearing mouse, possibly be attributed from the more robust immune system and the higher tolerance of C57BL/6 mice [51]. Additionally, mHFn@MTO plus laser treatment resulted in the lowest excised tumor weights (Fig. 6E), further supporting the superiority of mHFn@MTO plus laser for tumor treatment. Importantly, mHFn@MTO alone or combined with laser caused no obvious decrease in body weight of tumor-bearing C57BL/6 mice, whereas MTO and MTO plus laser groups induced gradual decrease in the body weight (Fig. 6F). The H&E staining showed that no obvious morphological or pathological changes were observed in the main organs (heart, liver, spleen, lung, and kidney) with all treatments (Fig. S16), indicating that the treatments did not cause significant damage to the examined organs. Furthermore, the levels of liver function indices (ALT, AST, and AKP) and kidney function indices (CRE and BUN) in serum all remained within the reference values after different treatments (Fig. S17). All these results verified the potential of mHFn@MTO for synergistic chemo-photothermal therapy to eradicate tumors while maintaining good biosafety.

## Conclusion

In this study, we exploited a pH and thermal-responsive mHFn protein nanocage for tumor targeted delivery and laser-controlled release of MTO to promote the synergistic chemo-photothermal therapy of colorectal cancer. Due to the inherent affinity of mHFn to TfR, MTO was efficiently and specifically delivered to tumor tissues, resulting in high tumor accumulation. Upon laser irradiation, MTO was rapid released from the opened thermal-sensitive drug entry channels of mHFn nanocage, which further facilitated the deep tumor penetration. By inducing a large amount of ROS generation under laser irradiation and then disrupting mitochondrial function, mHFn@MTO significantly induced tumor cells apoptosis and inhibited tumor growth without the adverse side effects typically associated with traditional chemotherapy, leading to tumor free in 2/6 CT26 and MC38 tumor-bearing mice. With its effective MTO loading capacity and efficient laser-responsive drug release, mHFn@MTO holds promise as a candidate for chemo-photothermal combination therapy against colorectal cancer.

### Supplementary Information

Additional file 1: **Figure S1.** PDI and zeta potential of mHFn and mHFn@MTO nanocage in Tris buffer. **Figure S2.** Hydrodynamic size, PDI and zeta potential of mHFn@MTO nanocage in Tris or PBS with 10% FBS. **Figure S3.** The particle size changes of mHFn@MTO at different pH and different temperature. **Figure S4.** The quantification and flow charts of TfR expression on NIH3T3, CT26 and MC38 cells. **Figure S5.** The cellular uptake of mHFn@MTO in the presence and absence of anti-TfR in NIH3T3 cells. **Figure S6.** Uptake and subcellular localization of free MTO in CT26 cells. **Figure S7.** Uptake and subcellular localization of mHFn@MTO in CT26 cells. **Figure S8.** The fluorescence microscope images of total ROS accumulation in CT26 cells. **Figure S9.** FACS analysis of mitochondrial membrane potential in CT26 cells. **Figure S10.** The fluorescence microscope images of mitochondrial membrane potential in CT26 cells. **Figure S11.** FACS analysis of cell apoptotic percentage in CT26 cells. **Figure S12.** FACS analysis of cell cycle in CT26 cells. **Figure S13.** Viability of MC38 cells after incubation with different formulations. **Figure S14.** Cell viability data in CT26 cells and MC38 cells after the treatment of mHFn. **Figure S15.** The quantification of MTO accumulation in different organs and tumors at different timepoints post intravenous injection. **Figure S16.** H&E staining of major healthy organs at the end of vivo efficacy study. **Figure S17.** Serum biochemistry analysis at the end of the* in vivo *experiment. **Table S1.** Half maximal inhibitory concentration (IC_50_) of MTO in different groups in CT26 cells and MC38 cells.

## Data Availability

All data are available in the main text or the supplementary materials. Data for all conditions are presented in the supplementary material.

## Abbreviations

mHFn: Murine heavy chain ferritin
TfR: Transferrin receptor
ROS: Reactive oxygen species
CRC: Colorectal cancer
PTT: Photothermal therapy
MTO: Mitoxantrone
SDS-PAGE: Sulfate-polyacrylamide gel electrophoresis
BCA assay: Bicinchoninic acid assay
PDI: Polydispersity index
DLS: Dynamic light scattering
PBS: Phosphate buffer solution
ALT: Alanine aminotransferase
AST: Aspartate aminotransferase
AKP: Alkaline phosphatase
CRE: Creatinine
BUN: Urea nitrogen
