# Supplementary material for: Laser-activable murine ferritin nanocage for chemo-photothermal therapy of colorectal cancer
Source: J Nanobiotechnology. 2024 May 29;22:297. doi: 10.1186/s12951-024-02566-6 (PMC11134727; doi:10.1186/s12951-024-02566-6)
Supplement: Supplementary file 1 — Additional file 1: Figure S1. PDI and zeta potential of mHFn and mHFn@MTO nanocage in Tris buffer. Figure S2. Hydrodynamic size, PDI and zeta potential of mHFn@MTO nanocage in Tris or PBS with 10% FBS. Figure S3. The particle size changes of mHFn@MTO at different pH and different temperature. Figure S4. The quantification and flow charts of TfR expression on NIH3T3, CT26 and MC38 cells. Figure S5. The cellular uptake of mHFn@MTO in the presence and absence of anti-TfR in NIH3T3 cells. Figure S6. Uptake and subcellular localization of free MTO in CT26 cells. Figure S7. Uptake and subcellular localization of mHFn@MTO in CT26 cells. Figure S8. The fluorescence microscope images of total ROS accumulation in CT26 cells. Figure S9. FACS analysis of mitochondrial membrane potential in CT26 cells. Figure S10. The fluorescence microscope images of mitochondrial membrane potential in CT26 cells. Figure S11. FACS analysis of cell apoptotic percentage in CT26 cells. Figure S12. FACS analysis of cell cycle in CT26 cells. Figure S13. Viability of MC38 cells after incubation with different formulations. Figure S14. Cell viability data in CT26 cells and MC38 cells after the treatment of mHFn. Figure S15. The quantification of MTO accumulation in different organs and tumors at different timepoints post intravenous injection. Figure S16. H&E staining of major healthy organs at the end of vivo efficacy study. Figure S17. Serum biochemistry analysis at the end of the in vivo experiment. Table S1. Half maximal inhibitory concentration (IC50) of MTO in different groups in CT26 cells and MC38 cells. [file 12951_2024_2566_MOESM1_ESM.docx]

**Additional file 1**

**Laser-activable murine Ferritin nanocage for chemo-photothermal therapy of colorectal cancer**

Jinmei Cheng^1^, Jiaxin Li^1^, Qilin Yu^1^, Peishan Li^1^, Junyi Huang^2^, Jinhui Li^3^, Leyang Guan^3^, Zhiyong Xu^1^, Jisheng Xiao^1,2,^*, Xiaopin Duan^1,^*

^1^Department of General Surgery, Zhujiang Hospital; Cancer Research Institute, School of Basic Medical Sciences, Southern Medical University, Guangzhou, Guangdong, 510515, China.

^2^Department of Cardiology, Heart Center; Department of Pharmacy; Guangdong Provincial Biomedical Engineering Technology Research Center for Cardiovascular Disease; Translational Medicine Research Center, Zhujiang Hospital, Southern Medical University, Guangzhou 510280, China.

^3^Experimental Education/Administration Center, School of Basic Medical Science, Southern Medical University, Guangzhou 510515, China.

Jinmei Cheng, Jiaxin Li and Qilin Yu are contributed equally to this work as first co‑authors.

*Correspondence: [jsx031@smu.edu.cn](mailto:jsx031@smu.edu.cn) (Jisheng Xiao) and [xpduan@smu.edu.cn](mailto:xpduan@smu.edu.cn) (Xiaopin Duan)

A full list of author information is available at the end of the article.

*List of Supplementary Materials:*

Figure S1. PDI and zeta potential of mHFn and mHFn@MTO nanocage in Tris buffer.

Figure S2. Hydrodynamic size, PDI and zeta potential of mHFn@MTO nanocage in Tris or PBS with 10% FBS.

Figure S3. The particle size changes of mHFn@MTO at different pH and different

temperature.

Figure S4. The quantification and flow charts of TfR expression on NIH3T3, CT26

and MC38 cells.

Figure S5. The cellular uptake of mHFn@MTO in the presence and absence of anti-TfR in NIH3T3 cells.

Figure S6. Uptake and subcellular localization of free MTO in CT26 cells.

Figure S7. Uptake and subcellular localization of mHFn@MTO in CT26 cells.

Figure S8. The fluorescence microscope images of total ROS accumulation in CT26 cells.

Figure S9. FACS analysis of mitochondrial membrane potential in CT26 cells.

Figure S10. The fluorescence microscope images of mitochondrial membrane potential in CT26 cells.

Figure S11. FACS analysis of cell apoptotic percentage in CT26 cells.

Figure S12. FACS analysis of cell cycle in CT26 cells.

Figure S13. Viability of MC38 cells after incubation with different formulations. Figure S14. Cell viability in CT26 cells and MC38 cells after the treatment of mHFn.

Figure S15. The quantification of MTO accumulation in different organs and tumors at different timepoints post intravenous injection.

Figure S16. H&E staining of major healthy organs at the end of vivo efficacy study. Figure S17. Serum biochemistry analysis at the end of the *in vivo* experiment.

Table S1. Half maximal inhibitory concentration (IC_50_) of MTO in different groups in CT26 cells and MC38 cells.


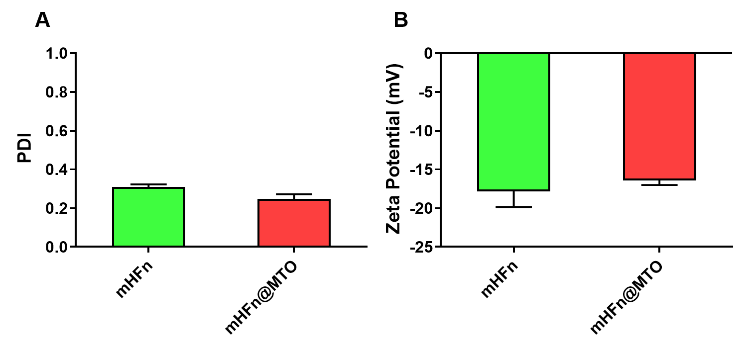


**Figure S1.** (A) PDI and (B) zeta potential of mHFn and mHFn@MTO nanocage in Tris buffer.


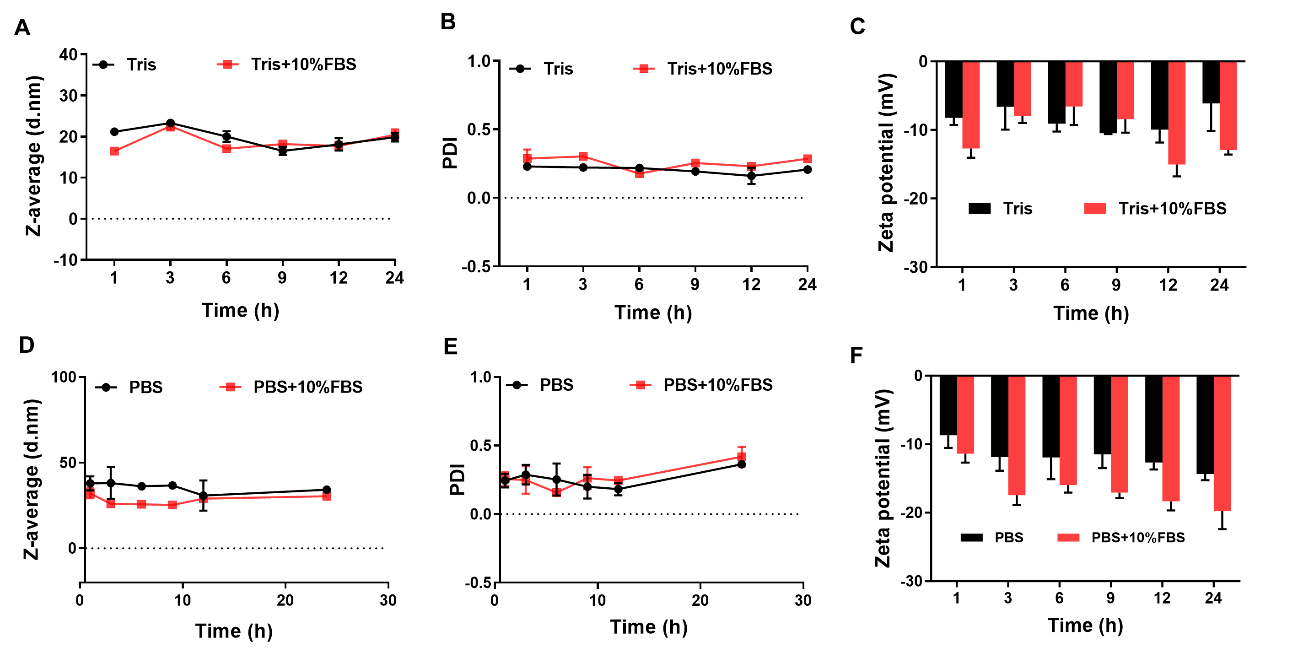


**Figure S2.** The changes of (A, D) hydrodynamic size, (B, E) PDI and (C, F) zeta potential of mHFn@MTO after incubation in (A-C) Tris or (D-F) PBS with or without 10% FBS.


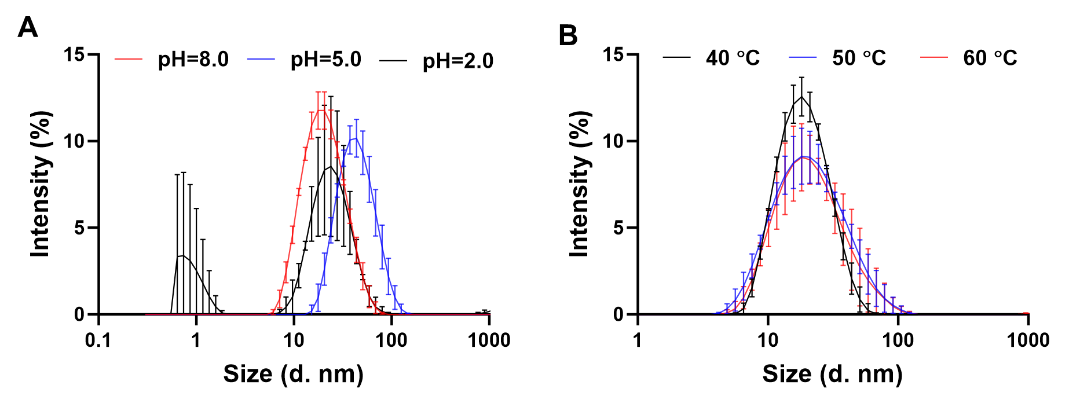


**Figure S3.** The particle size changes of mHFn@MTO at (A) different pH and (B) different temperature.


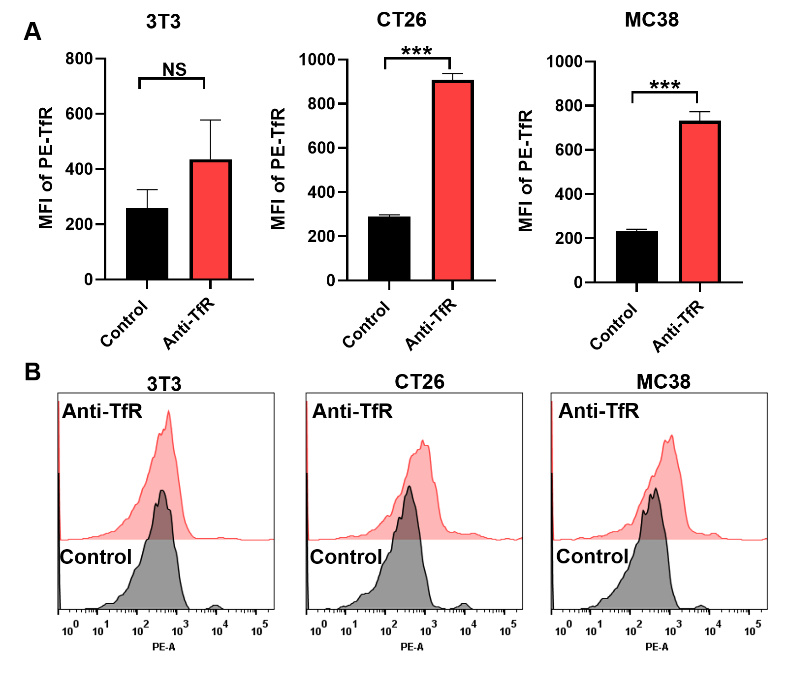


**Figure S4.** (A) The quantification and (B) flow charts of TfR expression on NIH3T3, CT26, and MC38 cells.


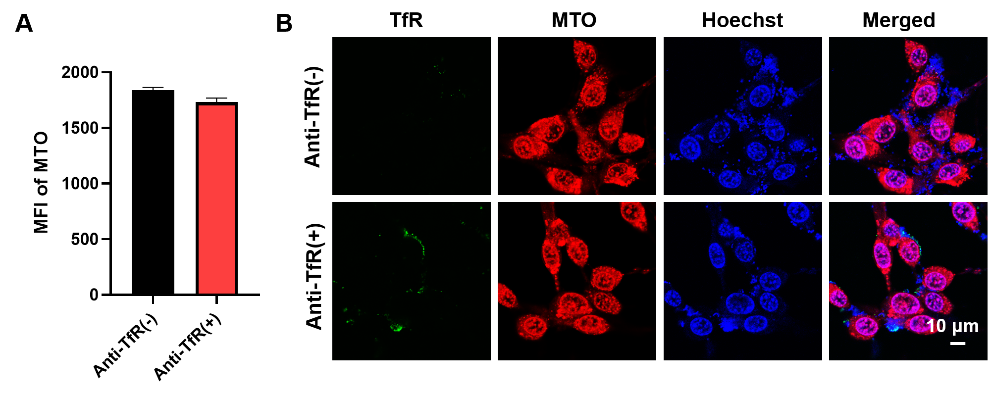


**Figure S5.** (A) The cellular uptake of mHFn@MTO in the presence and absence of anti-TfR in NIH3T3 cells, determined by flow cytometry. (B) The CLSM images showing TfR expression and mHFn@MTO internalization in NIH3T3.


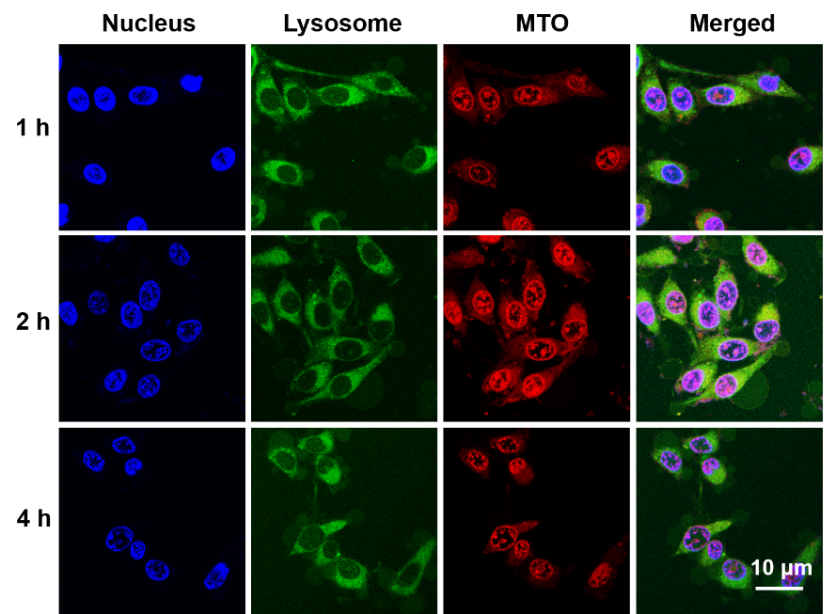


**Figure S6.** Uptake and subcellular localization of free MTO in CT26 cells observed by CLSM at different timepoints (1, 2, and 4 h).


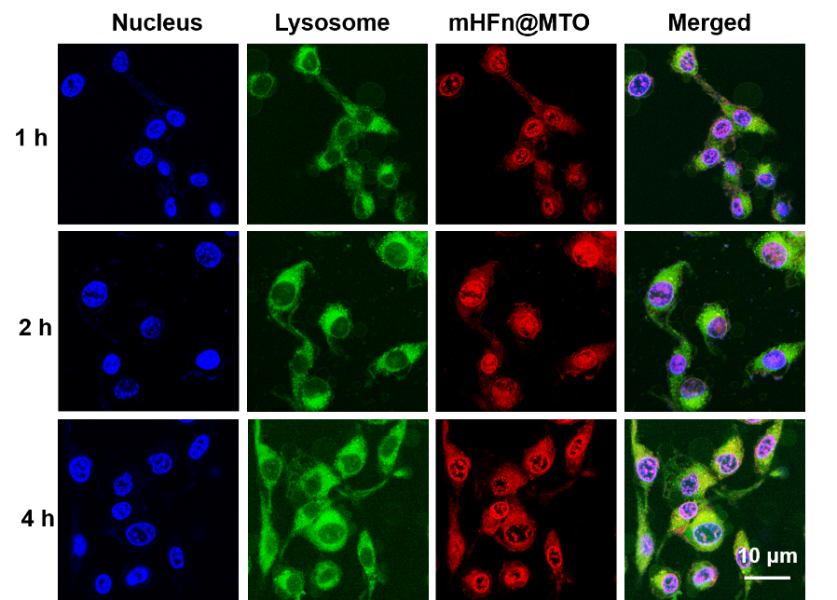


**Figure S7.** Uptake and subcellular localization of mHFn@MTO in CT26 cells observed by CLSM at different timepoints (1, 2, and 4 h).


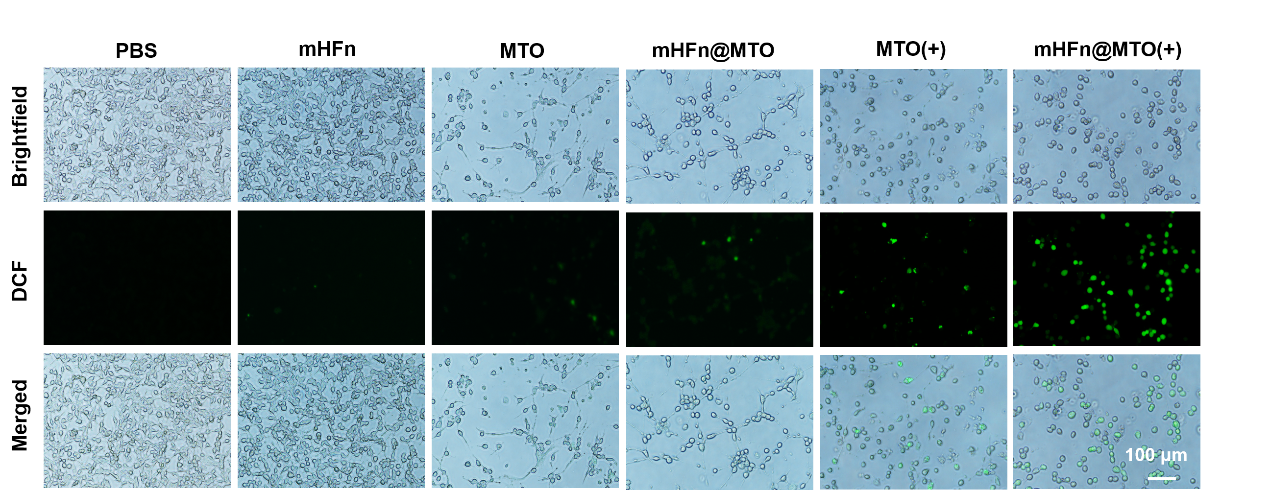


**Figure S8.** The fluorescence microscope images of total ROS accumulation in CT26 cells after different treatments.


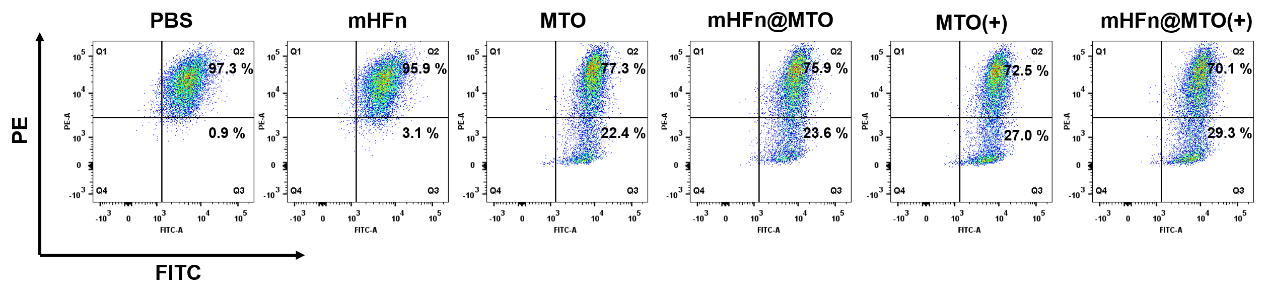


**Figure S9.** FACS analysis of mitochondrial membrane potential in CT26 cells after different treatments.


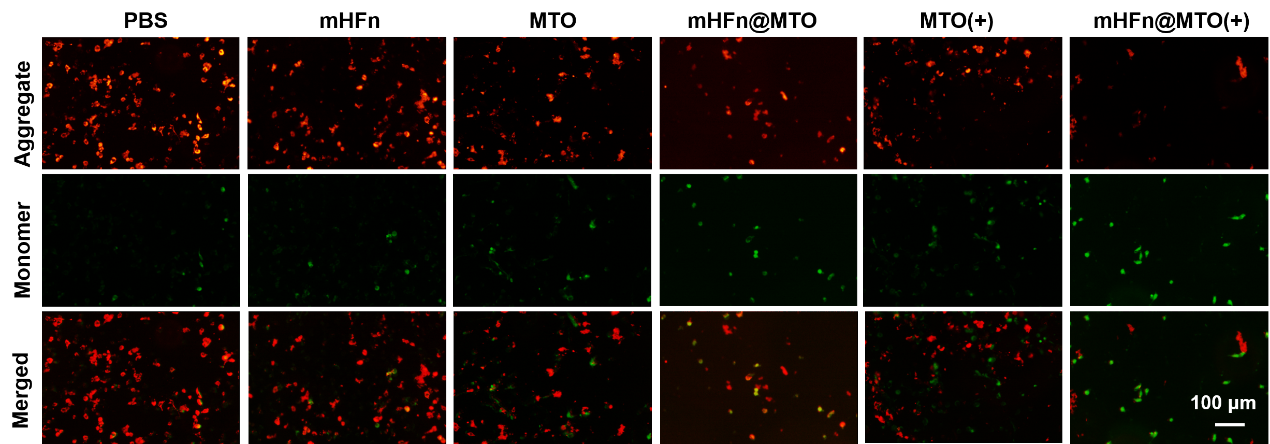


**Figure S10.** The fluorescence microscope images of mitochondrial membrane potential in CT26 cells after different treatments.


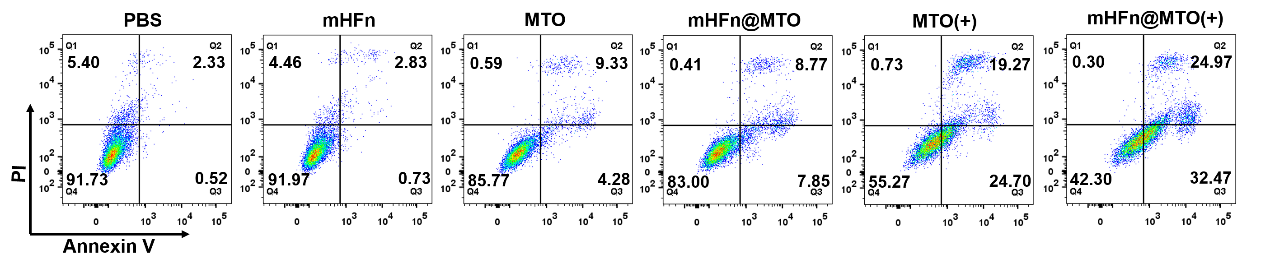


**Figure S11.** FACS analysis of cell apoptotic percentage in CT26 cells incubated with different formulations.


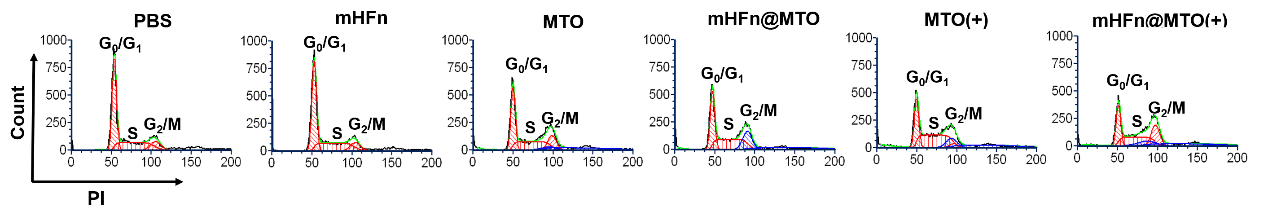


**Figure S12.** FACS analysis of cell cycle in CT26 cells treated with different formulations.


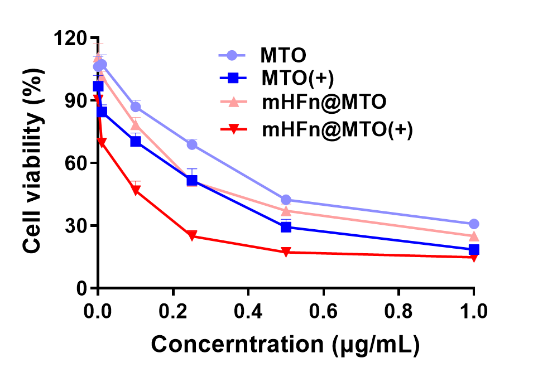


**Figure S13.** Viability of MC38 cells after incubation with various concentrations of MTO and mHFn@MTO with or without laser irradiation.


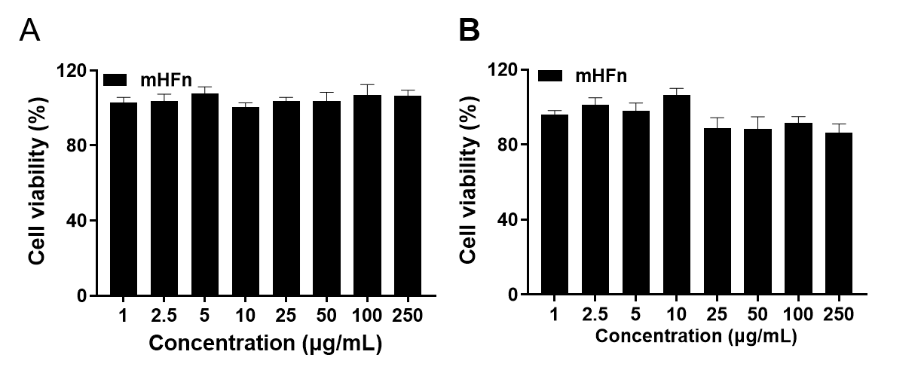


**Figure S14.** Cell viability data in (A) CT26 cells and (B) MC38 cells after the treatment of mHFn for 48 h.


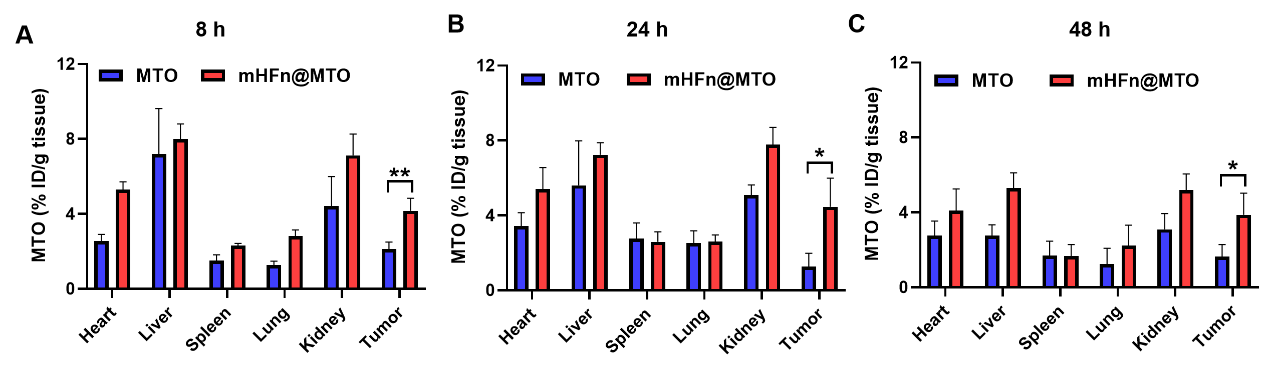


**Figure S15.** The quantification of MTO accumulation in different organs and tumors at (A) 8 h, (B) 24 h and (C) 48 h.


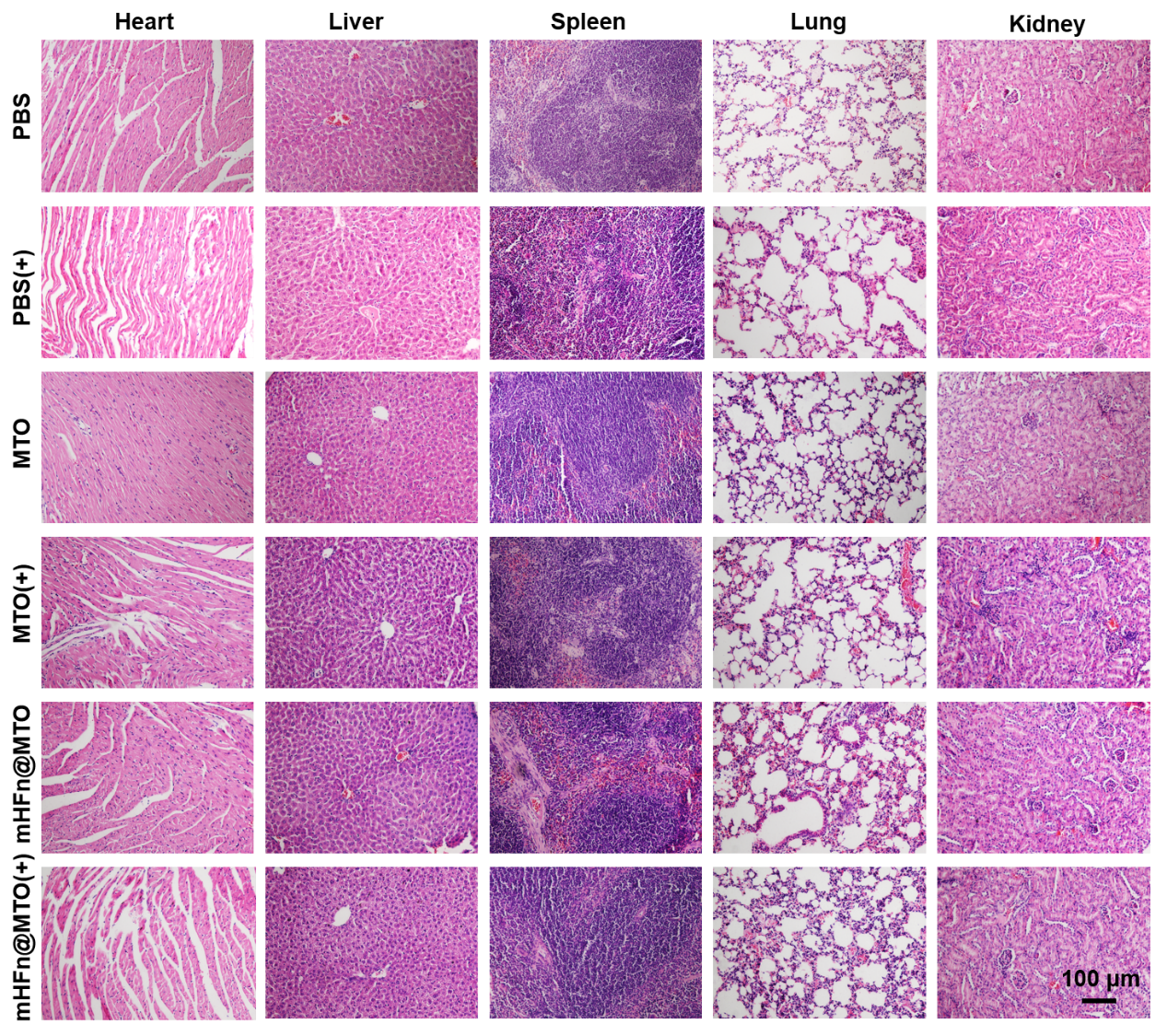


**Figure S16.** H&E staining of major healthy organs (heart, liver, spleen, lung, and kidney) at the end of vivo efficacy study in response to different treatments.


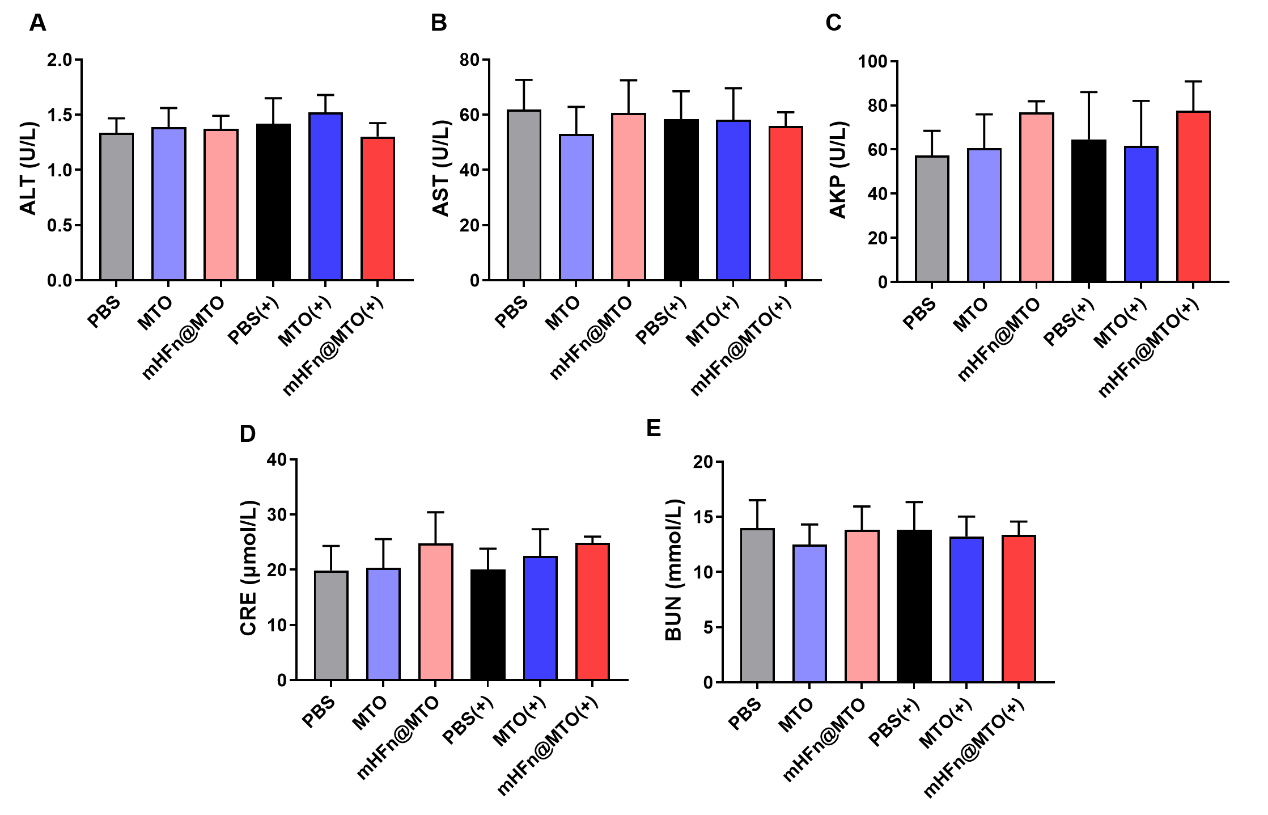


**Figure S17.** Serum biochemistry analysis of (A) ALT, (B) AST, (C) AKP, (D) CRE, and (E) BUN at the end of the *in vivo* experiment.

**Table S1.** Half maximal inhibitory concentration (IC_50_) of MTO in different groups in CT26 cells and MC38 cells.

| IC_50_ (μg/mL) | CT26 cells | MC38 cells |
| --- | --- | --- |
| MTO | 0.32 ± 0.04 | 0.52 ± 0.05 |
| mHFn@MTO | 0.12 ± 0.01 | 0.32 ± 0.03 |
| MTO(+) | 0.13 ± 0.02 | 0.24 ± 0.02 |
| mHFn@MTO(+) | 0.08 ± 0.01 | 0.08 ± 0.01 |
